# A Robust Method to Store Complement C3 With Superior Ability to Maintain the Native Structure and Function of the Protein

**DOI:** 10.3389/fimmu.2022.891994

**Published:** 2022-05-03

**Authors:** Anna Adler, Vivek Anand Manivel, Karin Fromell, Yuji Teramura, Kristina N. Ekdahl, Bo Nilsson

**Affiliations:** ^1^ Rudbeck Laboratory, Department of Immunology, Genetics and Pathology (IGP), Uppsala University, Uppsala, Sweden; ^2^ Cellular and Molecular Biotechnology Research Institute (CMB), National Institute of Advanced Industrial Science and Technology (AIST), Tsukuba, Japan; ^3^ Linnaeus Center of Biomaterials Chemistry, Linnaeus University, Kalmar, Sweden

**Keywords:** C3, C3(H2O), thioester, storage, freezing

## Abstract

Complement components have a reputation to be very labile. One of the reasons for this is the spontaneous hydrolysis of the internal thioester that is found in both C3 and C4 (but not in C5). Despite the fact that ≈20,000 papers have been published on human C3 there is still no reliable method to store the protein without generating C3(H_2_O), a fact that may have affected studies of the conformation and function of C3, including recent studies on intracellular C3(H_2_O). The aim of this work was to define the conditions for storage of native C3 and to introduce a robust method that makes C3 almost resistant to the generation of C3(H_2_O). Here, we precipitated native C3 at the isoelectric point in low ionic strength buffer before freezing the protein at -80°C. The formation of C3(H_2_O) was determined using cation exchange chromatography and the hemolytic activity of the different C3 preparations was determined using a hemolytic assay for the classical pathway. We show that freezing native C3 in the precipitated form is the best method to avoid loss of function and generation of C3(H_2_O). By contrast, the most efficient way to consistently generate C3(H_2_O) was to incubate native C3 in a buffer at pH 11.0. We conclude that we have defined the optimal storage conditions for storing and maintaining the function of native C3 without generating C3(H_2_O) and also the conditions for consistently generating C3(H_2_O).

## Introduction

The complement system is initiated through three distinct pathways known as the classical pathway (CP), the lectin pathway (LP) and the alternative pathway (AP). Complement protein C3, was for the first time isolated in the 1960s by Müller-Eberhard et al. (1) and plays a central role as the first common complement components of all three pathways leading to activation of the terminal pathway. Despite the fact that today (spring 2022) ≈20,000 papers have been published on human C3, there is still no reliable method to store the protein without affecting the conformation and function of the protein, a fact that may affect the interpretation of many studies of C3, including recent studies on intracellular C3 activation (2, 3).

In the early 1980s, it was demonstrated that C3 (together with C4) contains a unique thioester bond essential for its covalent binding to target surfaces (4, 5). In the 1980s Pangburn et al. described the spontaneous hydrolysis of the thioester bond within C3 and called this form of C3 “C3(H_2_O)” (6–8). C3(H_2_O) is a hemolytic inactive product which displays a “C3b-like” conformation with an intact α-chain and the C3a domain still remaining (9). We, and others, have shown that an intermediate form of C3(H_2_O) exists which is conformationally different from both native C3 and C3(H_2_O) (7, 10). C3(H_2_O) is susceptible to cleavage by factor I in the presence of factor H and it can form an initial C3 convertase (8). C3(H_2_O) is generated at a slow rate, concomitant with loss of its hemolytic activity, and the hydrolysis of the internal thiol ester has been estimated to occur at a rate of 0.2-0.4% per hour in plasma (3, 11, 12). The formation of C3(H_2_O) can be facilitated by treatment of native C3 with e.g., chaotropic agents (e.g., potassium thiocyanate, and guanidine), at the interface of gas and fluid or by slowly freezing and thawing (F/T) C3. A similar form of C3 is also acquired by treating C3 with nucleophiles (e.g., ammonia, and methylamine) (3, 6, 11, 13).

Complement has a reputation of being very labile due to the spontaneous hydrolysis of the internal thioester of C3 and C4 (but not in C5 despite their great homology (13)), which causes a loss of function (e.g., hemolysis) in serum samples but also makes isolated C3 very sensitive to freezing. Today the most common method to store purified native C3 is to freeze it in a physiological buffer. However, when storing native C3 under these conditions it is very important to freeze the protein rapidly at -80°C or lower and to avoid repeated F/T cycles since the C3 loses its activity, due to the generation of C3(H_2_O). Studies of C3(H_2_O) have been accelerated due to its involvement in alternative pathway activation and to recent studies of intracellular complement activation (2, 14). To avoid heterogeneity with different conformational forms of C3, appropriate handling of C3 in order to control the protein under experimental conditions is essential (10). Careful characterization of C3(H_2_O) conformations would perhaps have resulted in other conclusions of several previous studies investigating the function and structure of C3(H_2_O). This is underlined by the fact that both maintaining C3 in its native form and the opposite to fully push C3 into its C3(H_2_O) form has been challenging.

The aim of this brief research report is to establish the optimal conditions for storage of native C3 and to introduce a method which protects C3 from being transferred into C3(H_2_O) in its purified form. Based on these studies, we also demonstrate how native C3 can be consistently turned into C3(H_2_O) without contamination with native C3.

## Material and Methods

CaCl_2_, HCl, KH_2_PO_4_, MgCl_2_, NaCl, and Na_2_HPO_4_ were purchased from Merck KGaA (Darmstadt, Germany). PBS tablets (0.14 M NaCl, 2.7 mM KCl, 10 mM phosphate buffer, pH 7.4) were obtained from Medicago AB (Uppsala, Sweden). Bovine serum albumin (BSA), methylamine hydrochloride, EDTA and gelatin was obtained from Sigma Aldrich (St Louis, MO, USA). Buffers were prepared as follows: VB^++^ (Veronal-buffered saline) containing 5mM Na-barbiturate, pH 7.4 (145 mM NaCl; 0.15 mM Ca^2+^; 0.5 mM Mg^2+^), GVB^++^ (VB^++^ with 0.5% w/v gelatin), buffer A for Mono S, 20 mM PBS pH 6.8 (Na_2_HPO_4_ 2.054 g + KH_2_PO_4_ 1.151 g + 350 µL 37% HCl in 1L MQ-H_2_O), buffer B for Mono S, 20 mM PBS pH 6.8, and 1 M NaCl (Na_2_HPO_4_ 2.054 g + KH_2_PO_4_ 1.151 g + 350 µL 37% HCl + 58.44 g NaCl in 1L MQ-H_2_O). C3-depleted serum and C3b were purchased from Complement Technology (Tyler, TX, USA). The mAb 4SD17.3, as previously described (15–17), which binds to a neoepitope in free C3a, as well as to the C3a moiety in C3(H_2_O), was used as a coating antibody in the C3(H_2_O) ELISA. Anti-human C3d obtained from Dako (A0064, Glostrup, Denmark), biotinylated in-house (18), was used as the detection antibody in the C3(H_2_O) ELISA. Streptavidin-HRP was obtained from GE Healthcare (RPN1231, Uppsala, Sweden). TMB (3,3´,5,5´-tetramethybenzidine) was purchased from Surmodics (Minnesota, USA).

### Purification and Storage of Native C3

Native C3 was purified in-house from human plasma according to Hammer et al. (19). After the final step of purification, C3 was precipitated in a low salt and pH buffer for storage by dialysis against a 40 mM phosphate buffer with ionic strength 0.05 (mS) and pH 6.0 at +4°C with a buffer volume at least 10-fold higher than the volume of C3. After 24 h the buffer was replaced with fresh buffer and dialysis continued for another 24 h until a visible precipitate was formed. The stock solution of precipitated native C3 was then suspended (without centrifugation) and aliquoted before storage at -80°C. In order to dissolve the precipitated C3, 1-part VB^++^ (5x stock solution) was added to 4-parts of precipitated C3 immediately before use.

### C3 Preparations

The samples were prepared as described in **Table 1**. The C3 called “C3 precipitate” was F/T in the precipitated form and “native C3” is C3 precipitate that has been dissolved, as described above, before being subjected to F/T cycles, methylamine, or different pH conditions. All samples were prepared in 0.5 mL fractions at concentrations of 0.7 mg/mL C3. Slow F/T is defined as F/T from -20°C to room temperature (RT), and fast F/T is defined as F/T from -80°C to 37°C. Note that before running the samples on the column, the precipitated forms were dissolved in VB^++^ stock (5x) to a concentration of 0.7 mg/mL. All the different C3 samples were buffer exchanged to buffer A using PD MiniTrap G-25 columns with the gravity protocol (GE Healthcare, Buckinghamshire, UK) before applying them on the Mono S column.

**Table 1:** Preparations of the different pure C3 samples analyzed by cation exchange chromatography.

Sample	Preparation*
**A**	Native C3, never frozen freshly dissolved C3 precipitate	
**B**	Native C3 incubated in 0.2 M methylamine in VB^++^ pH 8.0 for 30 min at 37°C	
**C**	Native C3 incubated in 0.5 M methylamine in VB^++^ pH 8.0 for 30 min at 37°C	
**D**	C3 precipitate slow F/T from -20°C to RT x5	
**E**	Native C3 slow F/T from -20°C to RT x1	
**F**	Native C3 slow F/T from -20°C to RT x5	
**G**	C3 precipitate fast F/T from -80°C to 37°C x5	
**H**	Native C3 fast F/T from -80°C to 37°C x1	
**I**	Native C3 F/T from -80°C to 37°C x5	
**J**	Native C3 treated in VB^++^ pH 5.0 for 30 min at 37°C	
**K**	Native C3 treated in VB^++^ pH 8.0 for 30 min at 37°C	
**L**	Native C3 treated in VB^++^ pH 11.0 for 30 min at 37°C	

*F/T, freeze/thaw; RT, room temperature.

### Cation Exchange Chromatography

The chromatography was performed using a Mono S 5/50 GL column (GE Healthcare, Bio-Sciences AB, Uppsala, Sweden) with a column volume of 1.0 mL and coupled to the NGC™ Chromatography System (BioRad, USA). The flow rate was set to 0.5 mL/min at RT throughout the whole separation process. Briefly, the column was first equilibrated with 5 column volumes (CV) buffer A (20 mM phosphate buffer pH 6.8), followed by sample application (0.25 CV). The column was washed with 3 CV of buffer A and then the elution was started using a linear gradient of buffer B ranging from 0 to 0.85M NaCl in 20 mM phosphate buffer pH 6.8 (total volume 17.9 CV). The protein elution was monitored with an UV detector at 280 nm and fractions of 0.5 mL were collected during the elution procedure.

### Hemolytic Assay for the Classical Pathway

A hemolytic assay for CP was performed to investigate the hemolytic activity of C3 treated in pH 11.0, slow and fast F/T C3 precipitate or native C3. Freshly dissolved native C3 was used as a positive control and to create a standard curve and C3b was used as a negative control. First, sheep erythrocytes were sensitized with IgM according to Nilsson and Nilsson (20). A standard curve was made by spiking freshly dissolved native C3, ranging from 12.5-0.008 µg/mL C3, into GVB^++^ with 5 µL C3-depleted serum (Complement technology) to a final volume of 500 µL. C3-depleted sera in GVB^++^ without the addition of C3 was used as a blank. The different C3 preparations were spiked into diluted C3-depleted sera in GVB^++^ to a final concentration of 0.4 µg/mL C3 and a final volume of 500 µL. Then, 500 µL of 4 x 10^7^ IgM sensitized sheep erythrocytes/mL (final concentration; 0.05% sheep erythrocytes) were added to each tube and incubated at 37°C on a plate shaker (600 rpm) for 45 min. For total lysis 1 mL of pure water was added to 4 x 10^7^ cells/mL. The reaction was stopped with 1 mL of cold 10 mM EDTA in VB^++^. The samples were centrifuged at 4°C for 10 min at 860*g*. Thereafter, 250 µL of the supernatant, in duplicate, were transferred into a 96-well microtiter plate and the absorbance was measured at 414 nm. All samples were run in quadruplicate. The absorbance from the blank was removed from the samples and the % of lysis was calculated based on the total lysis.

### Blood Collection and Sample Preparation

Blood was collected from six healthy donors who had not received any medication for a minimum of 10 days prior to donation. For preparation of EDTA plasma, human whole blood was collected in Vacuette^®^ tubes (Greiner Bio-One GmbH, Kremsmünster, Austria) containing K2EDTA (4 mM final concentration) and centrifuged at RT for 15 min at 2000*g*. Ethical approval to use human blood was obtained from the regional ethics board in Uppsala (diary nr 2008/264). The plasma was aliquoted into 100 µL aliquots and stored either at +4°C for 2h (referred to as fresh plasma), or subjected to 1, 3 or 5 F/T cycles, from either -20°C to RT or from -80°C to 37°C, before being analyzed for the generation of C3(H_2_O) using ELISA.

### C3(H_2_O) ELISA

To analyze the generation of C3(H_2_O) in plasma subjected to repeated F/T cycles, an in-house C3(H_2_O) ELISA was used (11). All washing steps were performed three times with the wash buffer PBS 0.05% Tween 20 and PBS 0.05% Tween20 with 10 mM EDTA and 1% BSA was used as blocking and dilution buffer. All incubations steps were performed at RT on a plate shaker at 700 rpm. In brief, a 96-well Nunc Maxisorp plate (Thermo Fisher Scientific, Denmark) was coated with 100 µL of 0.8 µg/mL mAb anti-C3a 4SD17.3 in PBS overnight at 4°C. The plate was washed and subsequently blocked with 200 µL blocking buffer for 60 min and then was washed again. Plasma samples were diluted 1/200 and added in duplicate to the plate (100 µL/well) and incubated for 60 min. For the standard curve, pooled plasma from all donors were diluted 1/200 and spiked with 0.5-0.03 µg/mL C3 treated in VB^++^, pH 11.0 (prepared as described in **Table 1**), to represent C3(H_2_O). The plate was washed, followed by the addition of 100 µL 1 µg/mL of the biotinylated detection antibody anti-human C3d (Dako) for 60 min. After washing the plate, 100 µL of streptavidin-HRP (GE Healthcare Uppsala, Sweden) diluted 1/500 was added for 15 min. The plate was again washed followed by the addition of 100 µL TMB. Finally, the reaction was stopped using 100 µL 1M H_2_SO_4_ and the absorbance was measured at 450 nm.

### Statistical Analysis

Data are presented as mean ± SD or as representative images. Statistical calculations using Freidman’s tests followed by Dunn’s multiple comparison tests were performed using GraphPad Prism 9 for macOS (GraphPad Software, La Jolla, CA, USA). A *p*-value <0.05 was considered significant.

## Results

**Figure 1:**
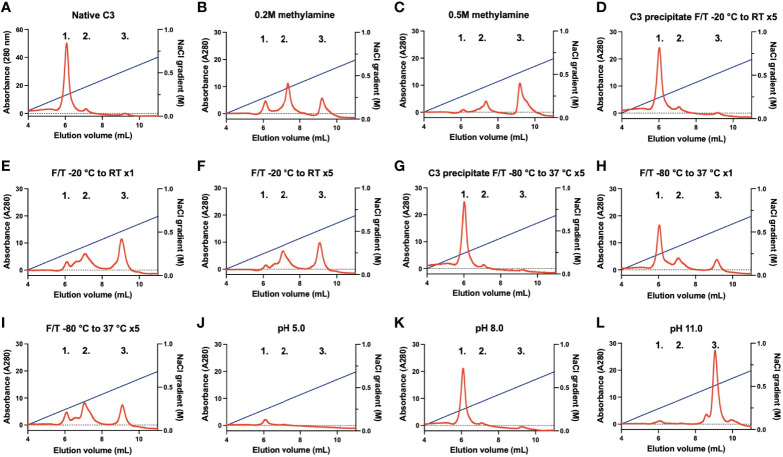
Representative chromatograms of different C3 samples analyzed on a Mono S column eluted with a gradient of 0-0.85 M NaCl. **Peak 1**; native C3, **peak 2**; C3(H_2_O) intermediate, and **peak 3**; C3(H_2_O). **(A)** native C3, **(B)** native C3 treated with 0.2 M methylamine, **(C)** native C3 treated with 0.5 M methylamine, **(D)** C3 in precipitated form F/T five times from -20°C to RT, **(E)** native C3 stored frozen once at -20°C **(F)** native C3 F/T five times from -20°C to RT, **(G)** C3 in precipitated form F/T five times from -80°C to 37°C, **(H)** native C3 frozen once at -80°C, **(I)** native C3 F/T five times from -80°C to 37°C, **(J)** native C3 treated at pH 5.0, **(K)** native C3 treated at pH 8.0 and **(L)** C3 treated at pH 11.0. F/T, freeze/thaw; RT, room temperature.

Native C3, C3(H_2_O) intermediate, and C3(H_2_O) are conformationally different and can be separated using cation exchange chromatography. In our set-up using a Mono S column, native C3 was eluted at 6 mL (0.25 M NaCl; peak 1), C3(H_2_O) intermediate at an estimated 7 mL (0.35 M NaCl; peak 2), and C3(H_2_O) at an estimated 9.5 mL (0.55 M NaCl; peak 3) (**Figure 1A**). Native C3 contained minute amounts of C3(H_2_O) intermediate and C3(H_2_O). The elution profiles of the methylamine treated C3 corresponded to the C3(H_2_O) intermediate and C3(H_2_O) peaks (**Figures 1B, C**). The more methylamine used, the more of the native C3 had been converted to C3(H_2_O), which was eluted in the last peak (0.2 M compared to 0.5 M). C3 in its precipitated form subjected to five slow F/T cycles (-20°C to RT) (**Figure 1D**) did not generate any C3(H_2_O). Whereas native C3 which had been frozen once at -20°C (**Figure 1E**) and native C3 subjected to five slow F/T cycles (-20°C to RT) (**Figure 1F**) only had a low level (a few percentage points) of native C3 remaining (peak 1). Precipitated C3 subjected to five fast F/T cycles (-80°C to 37°C) (**Figure 1G**) did not generate any C3(H_2_O). Native C3 which had been frozen once at -80°C (**Figure 1H**) had native C3 left, however the C3(H_2_O) intermediate and C3(H_2_O) peaks were more prominent compared to the freshly dissolved native C3 and the F/T precipitated C3. Native C3 subjected to five fast F/T cycles (-80°C to 37°C) (**Figure 1I**) had very little (less than 1%) native C3 left and were mainly in the C3(H_2_O) intermediate and C3(H_2_O) form. Native C3 subjected to a low pH (pH 5.0) (**Figure 1J**) seemed to be stuck in the spin-column membrane, therefore, no C3 is detected, thus indicating that this form of C3 is different. Treating C3 at pH 8.0 for 30 min at 37°C did not generate C3(H_2_O) (**Figure 1K**). This was also a control for the methylamine treated C3 since it was treated with methylamine in a VB^++^ buffer at pH 8.0. However, treating native C3 at pH 11.0 for 30 min at 37°C clearly pushed all native C3 to the C3(H_2_O) fraction (**Figure 1L**).

**Figure 2:**
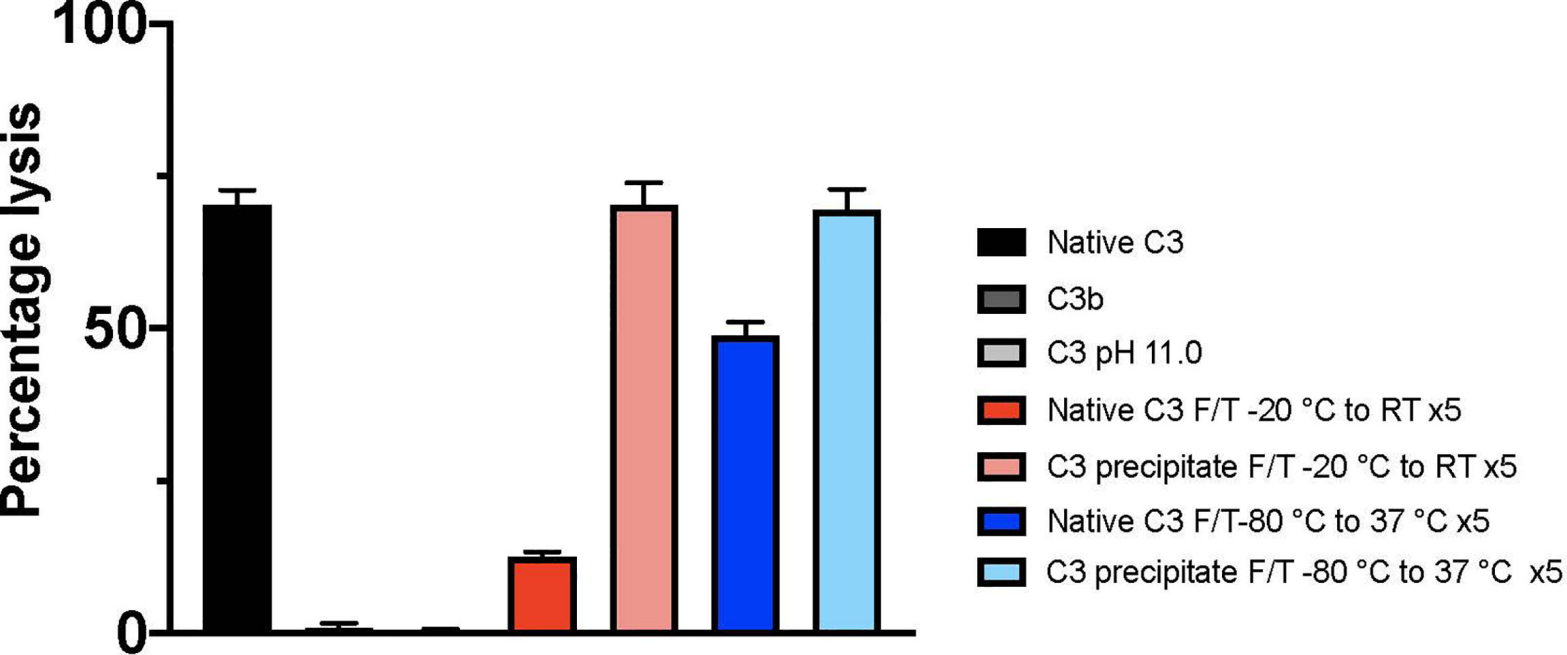
Hemolytic activity of different C3 preparations (identified by the panel designations in **Figure 1** and **Table 1**) analyzed by a hemolytic assay of the classical pathway. The lysis of 0.05% IgM sensitized erythrocytes, i.e., the hemolytic activity of C3, was determined by adding the cells into C3-depleted serum spiked with 0.4 µg/mL of native C3 (A), C3b, pH 11- treated C3 (L) or different preparations of F/T C3; precipitated and native C3 subjected to five F/T cycles from -20°C to RT (D, F) and C3 subjected to five F/T cycles from -80°C to 37°C (I, G), respectively. Total lysis was obtained by lysing the cells in pure water. The absorbance was measured at 414 nm. All samples were run in quadruplicates (n = 4). F/T, freeze/thaw; RT, room temperature.

The hemolytic assay for the CP (**Figure 2**) indicated that 0.4 µg/mL freshly dissolved native C3 spiked into C3-depleted serum had a hemolytic activity of 70.3% ± 2.1%, whereas C3b had no hemolytic activity (0.9% ± 0.7%). Native C3 treated in pH 11.0 was deprived of all hemolytic activity (0.5% ± 0.2%). C3 precipitate subjected to five slow or fast F/T cycles remained hemolytically active, 70.3% ± 3.1% and 69.4% ± 3.0%, respectively. Native C3 subjected to five slow or fast F/T cycles had a decreased hemolytic activity, 12.5% ± 0.8% and 48.9% ± 1.9%, respectively.

**Figure 3:**
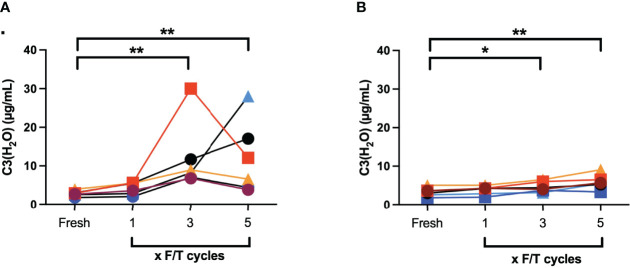
Quantification of C3(H_2_O) by ELISA in EDTA plasma (n = 6) that were subjected to repeated F/T cycles: **(A)** -20°C to RT; **(B)** -80°C to 37°C. *: *p*<0.05; **: *p*<0.001.

EDTA plasma samples from six different donors were subjected to repeated F/T cycles from -20°C to RT and from -80°C to 37°C and the generation of C3(H_2_O) was measured by ELISA. There was no difference in the C3(H_2_O) generation between fresh and plasma frozen once (**Figure 3**). However, when the plasma was F/T from -20°C to RT three times (12.1 ± 3.8 µg/mL, *p*=0.001) and five times (12.0 ± 3.8 µg/mL, *p*=0.005) there was an increase in C3(H_2_O) generation when compared with fresh plasma (3.3 ± 1.0 µg/mL). The corresponding values for -80°C to 37°C was for F/T three times (4.7 ± 1.2 µg/mL, *p*=0.042) and five times (5.9 ± 1.7 µg/mL, *p*=0.0010) compared with fresh plasma (2.8 ± 0.3 µg/mL).

## Discussion

### Purified C3

In this report we show that commonly used methods of storage of native C3 are detrimental for the protein conformation and function. This includes freezing and thawing of the protein at -20°C and -80°C in physiological buffer. From this we can conclude that soluble C3 preparations that are commercially available easily turn into C3(H_2_O) upon storage, especially after repeated F/T cycles, resulting in loss of hemolytic activity. Our investigation confirms that the best alternative at present is to quick-freeze and store the protein in liquid nitrogen in small aliquots avoiding repeated slow F/T of C3.

Our present data show that storage of purified native C3 precipitated at the isoelectric point, pH 5.7 (1), in a low ionic strength buffer, is the superior method to avoid loss of function and generation of C3(H_2_O) in the preparation. The precipitated form of C3 can be subjected to repeated slow and fast F/T cycles even at -20°C without generating C3(H_2_O), i.e., maintaining its full hemolytic activity. This precipitation method is unfortunately not applicable to storage of other thioester-containing proteins such as C4 (data not shown).

Native C3, C3(H_2_O) intermediate, and C3(H_2_O) have been shown to have distinctive conformations and properties (2). As shown in this study, the amount of native C3, C3(H_2_O) intermediate, and C3(H_2_O) in a sample depends on sample handling and storage. Therefore, when working with native C3/C3(H_2_O) it is important to know which conformations of C3 the preparation contains since it will affect the outcome of the experiments. In native C3, the positively charged C3a domain is hidden, therefore, when separated on the cation exchange chromatography native C3 elutes in the first peak followed by C3(H_2_O) intermediate and lastly C3(H_2_O). Similar elution profiles have been shown by others in previous studies (7, 10, 21).

In many publications, including those that initially defined C3(H_2_O) (6), it is obvious that not all of the C3 is transformed into C3(H_2_O) since some of the α-chain of C3 is still not susceptible to cleavage by factor I. Here, we present the optimal conditions for disrupting the thioester bond in order to consistently generate C3(H_2_O), which include subjecting native C3 to a physiological buffer and repeated F/T cycles at -20°C, and even more efficiently, incubating native C3 in a buffer at pH 11.0. At pH 11.0 almost 100% of the thioester bond is broken (22).

If the transfer to C3(H_2_O) is not complete the possibility still remains that C3(H_2_O) intermediate can go back to the native form of C3 as described by Pangburn et al. (7). In our previous study (10), we demonstrated that the C3(H_2_O) intermediate was more resistant to cleavage by FH and FI, while C3(H_2_O) is completely cleaved. We also showed that the intermediate is more efficient at creating a fluid phase initial C3 convertase of the AP compared to C3(H_2_O). Further studies of this form of C3 would highlight its possible role in the physiological function of C3.

### C3 in Plasma

In order to mimic the treatment of clinical patient samples and to investigate the C3(H_2_O) generation in plasma, we subjected EDTA plasma samples from six different donors to repeated F/T cycles. EDTA plasma was chosen to completely block further complement activation. Note that we are aware of that our “fresh” plasma stored at +4°C for 2h while performing the F/T cycles may have generated more C3(H_2_O) than initially existing in the blood. However, there is still an increase of C3(H_2_O) when the plasma F/T, and as also shown by Elvington et al., the generation of C3(H_2_O) when storing serum and plasma at +4°C appears to be minimal (3). The C3(H_2_O) ELISA indicated that C3(H_2_O) is generated in plasma when samples are subjected to repeated F/T cycles. This correlates well with what Elvington et al. also described while using another type of C3(H_2_O) ELISA (3). The implications are that when designing research experiments with plasma, it is important to be aware of this phenomenon. We also believe that the native C3 in plasma is protected by the surrounding plasma proteins from generating C3(H_2_O), therefore the C3(H_2_O) generation is not as serious as seen in the pure C3 preparations subjected to F/T as described above.

## Conclusions

In order to preserve the function of stored purified native C3, we have shown that native C3 kept in a precipitated form in a low-salt and low-pH buffer at -80°C is superior. Although not ideal, it can also be stored in this form at -20°C. By contrast, C3(H_2_O) is amply generated upon repeated F/T cycles under physiological conditions in both purified native C3 preparations and in human plasma, in particular, at -20°C but also at -80°C.

## Data Availability Statement

The original contributions presented in the study are included in the article/supplementary material. Further inquiries can be directed to the corresponding author.

## Author Contributions

BN, AA, and VM designed the research project. AA and VM performed the experiments. AA and BN wrote the manuscript with editorial assistance from VM, KF, KE, and YT. All authors participated in editing the final manuscript and have read and approved the final manuscript.

## Funding

The Swedish Research Council grants 2016-01060, 2016-04519, 2020-05762, and 2021-02252.

## Conflict of Interest

The authors declare that the research was conducted in the absence of any commercial or financial relationships that could be construed as a potential conflict of interest.

## Publisher’s Note

All claims expressed in this article are solely those of the authors and do not necessarily represent those of their affiliated organizations, or those of the publisher, the editors and the reviewers. Any product that may be evaluated in this article, or claim that may be made by its manufacturer, is not guaranteed or endorsed by the publisher.
